# Who Participates in Web-Assisted Tobacco Interventions? The Quit-Primo and National Dental Practice-Based Research Network Hi-Quit Studies

**DOI:** 10.2196/jmir.2385

**Published:** 2013-05-01

**Authors:** Rajani Shankar Sadasivam, Rebecca L Kinney, Kathryn Delaughter, Sowmya R Rao, Jessica Hillman Williams, Heather L Coley, Midge N Ray, Gregg H Gilbert, Jeroan J Allison, Daniel E Ford, Thomas K Houston, The National Dental PBRN And QUIT-PRIMO Collaborative Groups

**Affiliations:** ^1^Division of Health Informatics & Implementation ScienceUniversity of Massachusetts Medical SchoolWorcester, MAUnited States; ^2^VA eHealth Quality Enhancement Research InitiativeBedford VAMCBedford, MAUnited States; ^3^Center for Health QualityOutcomes & Economic Research (CHQOER)Bedford, MAUnited States; ^4^Department of Health Services AdministrationSchool of Health ProfessionsUniversity of Alabama at BirminghamBirmingham, ALUnited States; ^5^Division of General Internal MedicineUniversity of Alabama at BirminghamBirmingham, ALUnited States; ^6^Department of Clinical & Community SciencesSchool of DentistryUniversity of Alabama at BirminghamBirmingham, ALUnited States; ^7^Division of General Internal MedicineJohns Hopkins School of MedicineBaltimore, MDUnited States; ^8^University of Alabama at BirminghamBirmingham, ALUnited States

**Keywords:** smoking cessation, Web-assisted tobacco intervention, Google advertisements, medical practice, dental practice, public health informatics

## Abstract

**Introduction:**

Smoking is the most preventable cause of death. Although effective, Web-assisted tobacco interventions are underutilized and recruitment is challenging. Understanding who participates in Web-assisted tobacco interventions may help in improving recruitment.

**Objectives:**

To understand characteristics of smokers participating in a Web-assisted tobacco intervention (Decide2Quit.org).

**Methods:**

In addition to the typical Google advertisements, we expanded Decide2Quit.org recruitment to include referrals from medical and dental providers. We assessed how the expanded recruitment of smokers changed the users’ characteristics, including comparison with a population-based sample of smokers from the national Behavioral Risk Factors Surveillance Survey (BRFSS). Using a negative binomial regression, we compared demographic and smoking characteristics by recruitment source, in particular readiness to quit and association with subsequent Decide2Quit.org use.

**Results:**

The Decide2Quit.org cohort included 605 smokers; the 2010 BRFSS dataset included 69,992. Compared to BRFSS smokers, a higher proportion of Decide2Quit.org smokers were female (65.2% vs 45.7%, *P*=.001), over age 35 (80.8% vs 67.0%, *P*=.001), and had some college or were college graduates (65.7% vs 45.9%, *P*=.001). Demographic and smoking characteristics varied by recruitment; for example, a lower proportion of medical- (22.1%) and dental-referred (18.9%) smokers had set a quit date or had already quit than Google smokers (40.1%, *P*<.001). Medical- and dental-referred smokers were less likely to use Decide2Quit.org functions; in adjusted analysis, Google smokers (predicted count 17.04, 95% CI 14.97-19.11) had higher predicted counts of Web page visits than medical-referred (predicted count 12.73, 95% CI 11.42-14.04) and dental-referred (predicted count 11.97, 95% CI 10.13-13.82) smokers, and were more likely to contact tobacco treatment specialists.

**Conclusions:**

Recruitment from clinical practices complimented Google recruitment attracting smokers less motivated to quit and less experienced with Web-assisted tobacco interventions.

**Trial Registration:**

Clinicaltrials.gov NCT00797628; http://clinicaltrials.gov/ct2/show/NCT00797628 (Archived by WebCite at http://www.webcitation.org/6F3tqz0b3)

## Introduction

Smoking continues to be the number one preventable cause of death [1-5]. To meet the 2020 Healthy People objectives of ending the tobacco epidemic and reducing illness, disability, and death related to tobacco use and secondhand smoke exposure [6], innovative approaches are needed to reach and engage current smokers. Web-assisted tobacco interventions have improved quit rates [7-18] and can potentially reach a large number of smokers [19-21]. However, current methods to recruit smokers to these interventions have some limitations [22-26].

Most Web-assisted tobacco interventions recruit smokers by using search engine or media advertisements [27-28]. These methods require the smokers to actively initiate the first contact with the intervention; thus, the recruited smokers may not be representative of the majority of smokers [22-23], particularly in their readiness to quit. A Cochrane review of 20 Web-assisted tobacco interventions reported that most of these interventions that used search engine recruitment were able to recruit only those smokers ready to quit [29]. These smokers tended to be female and white [29-31]. Recruiting smokers by using just search engine or media advertisements also misses an important opportunity to recruit the majority of smokers, the 70% who see a health care provider at least once per year [3,5].

In our study, we expanded the recruitment of smokers to our evidence-based, Web-assisted tobacco intervention (Decide2Quit.org [32]) to also include provider referrals from medical and dental practices in addition to the usual Google advertisements. Our goal in this paper was to assess whether our expanded recruitment improved variability of our cohort. In particular, we were interested to see if directly recruiting smokers from provider’s e-referrals would increase participation of smokers less ready to quit. Finally, we looked longitudinally at these smokers’ participation in Decide2Quit.org; specifically, we looked at the association between recruitment source and subsequent use of Decide2Quit.org.

## Methods

### Study Design

We compared smokers who registered on Decide2Quit.org from May 2010 through July 2011 with smokers who responded to the 2010 BRFSS survey. Decide2Quit.org—a Web-assisted tobacco intervention containing information about quitting smoking, secure asynchronous messaging with a certified tobacco treatment specialist (TTS), an online support group, and a motivational, pushed-email, tailored messaging system—is the core patient intervention in 2 randomized trials. Decide2Quit.org is both a cessation induction and a relapse prevention system. All smokers can benefit from the system, whether they are in the precontemplation, contemplation, or preparation stage, or in the action or maintenance stages. Thus, we recruited smokers at all motivational levels. We used multiple routes to recruit smokers. In the Quality Improvement in Tobacco Provider Referrals & Internet-delivered Microsystem Optimization (Quit-Primo) trial, smokers were referred to Decide2Quit.org from medical practices [33] and in the Hygienists to Internet Quality Improvement in Tobacco (Hi-Quit) trial, smokers were referred from dental practices participating in a practice-based research network. In parallel, we also used Google advertisements to recruit smokers to Decide2Quit.org. These 2 trials were approved by the Institutional Review Boards of the University of Alabama at Birmingham and the University of Massachusetts Medical School.

The Behavioral Risk Factors Surveillance Survey (BRFSS) is a yearly, cross-sectional telephone survey conducted by state health departments with technical and methodological assistance provided by the Centers for Disease Control and Prevention to collect prevalence data on risk behaviors and preventive health practices that affect health status [34]. The health characteristics estimated from the BRFSS pertain to the adult population, aged 18 years or older. For our analysis, we used the 2010 BRFSS dataset because it was in the same time frame as the Decide2Quit.org registrations.

### Setting and Sample

The Decide2Quit.org cohort included smokers recruited from 81 medical primary care practices and 51 dental practices across the United States, and through Google advertisements. Primary care medical practices were recruited from a registered database of internal medicine and family/general practitioners. Dental practices were recruited from state lists of registered dentists and through the Dental Practice-Based Research Network [35]. At these practices, we implemented an e-referral program that allowed providers to recruit smokers to Decide2Quit.org at the point-of-care by entering their email addresses into an online form [33,36]. When e-referred, smokers were sent up to 10 email messages encouraging registration over an 8 week period or until the patient registered. To recruit smokers through Google advertisements, 3 ads were posted on Google AdWords [37]. Advertisements were linked to searches for keywords related to smoking (eg, smoking, quit smoking, stop smoking, quit, quit smoking tips, and quit smoking programs) and included a link that took participants directly to the Decide2Quit.org home page where they could choose to register as new participants.

The BRFSS is conducted monthly to collect data about risk behaviors from people in all 50 states, the District of Columbia, Puerto Rico, Guam, and the Virgin Islands. Respondents to the BRFSS were identified by using telephone-based methods; although 95% of US households have telephones, the coverage varies across states and subgroups. No direct methods were used to compensate for nontelephone coverage; however, post-stratification weights were used to partially correct for any bias caused by lack of telephone coverage. These weights also adjusted for differences in probability of selection and nonresponse. A more complete description of the sampling methodology may be found on the BRFSS website [34].

### Data Collection

All smokers registering on Decide2Quit.org completed an online survey during registration. We collected demographics (sex, age, ethnicity, education, marital status, and Internet usage) and smoking characteristics (readiness to quit, quit history, number of cigarettes per day, and smoking at home). Readiness to quit was assessed using a question based on the Transtheoretical Model of Change [38]. The readiness question consisted of 5 options: I am not thinking of quitting (precontemplation), I am thinking of quitting (contemplation), I have set a quit date (preparation), I quit today (action), and I have already quit (maintenance). Online activity (number of visits, page visits, asynchronous interactions with TTS, and use of the online support group) was tracked through Web page scripts.

The BRFSS questionnaire includes a standard set of questions asked by all states about current health-related perceptions, conditions, and behaviors, including smoking, as well as demographic questions. We used the question “Do you now smoke cigarettes every day, some days, or not at all?” to identify smokers in the 2010 BRFSS dataset.

### Statistical Analyses

All analyses were conducted using Stata version 11 (StataCorp LP, College Station, TX, USA). We first compared demographic characteristics of Decide2Quit.org smokers and BRFSS smokers. Next, we compared demographic and smoking characteristics by Decide2Quit.org recruitment source (Google advertisements vs clinical practice referrals). We also compared the readiness to quit of these smokers. We used the Pearson chi-square test to assess the significant differences between Decide2Quit.org and BRFSS smokers (survey weighted proportions). For each recruitment source, we assessed the number of referrals per successful smoker registration. For medical and dental practices, we divided the total referral count by the number of smokers subsequently registered in that group. For Google, we used the number of click throughs on our advertisement as the referral number and divided this number by the subsequent number of smokers registered in that group. This is an estimate of the number of referred smokers needed per successful registration. Finally, we assessed whether recruitment source was associated with subsequent use of the system. In this analysis, our dependent variable was use of the system measured by number of pages visited, our independent variable was recruitment source, and our covariates were demographics and smoking characteristics.

We used a count regression because our dependent variable was the number of pages visited. Because of over dispersion of the variance of the distribution of the dependent variable, we used negative binomial regression instead of simple Poisson. From these models, incidence rate ratios (IRR) and adjusted counts of the dependent variable were calculated. Although we primarily used number of pages visited as our system use variable, we also assessed use by using number of visits to the system.

## Results

### Summary

The Decide2Quit.org sample included 605 registered smokers: 32.6% (n=197) were from Google advertisements, 46.4% (n=280) were from medical practice e-referrals, and 21.0% (n=127) were from dental practice e-referrals. The 2010 BRFSS dataset included 69,992 smokers aged 18 and older.

### Decide2Quit.org Versus Behavioral Risk Factors Surveillance Survey

Compared with the national sample of smokers participating in the BRFSS, a higher proportion of Decide2Quit.org smokers were female (65.2% vs 45.7%, *P*=.001), over the age of 35 years (80.8% vs 67.0%, *P*=.001), and had attended some college or were a college graduate (65.7% vs 45.9%, *P*=.001) (Table 1). A small but significant difference was seen in the proportion of smokers who attempted to quit in the past 12 months (5%, *P*=.01).

### Number of Referrals Per Smoker Registration by Recruitment Source

The number of medical practice referrals was 1588 resulting in 280 successful registrations; thus, the number of referrals per registration was 5.7. The number of dental practice referrals was 739 with 127 registrations; the number of referrals per registration was 5.8. There were 6992 click throughs on our Google advertisements and a resulting 197 registrations; the number of referrals per registration was 35.5.

We compared Decide2Quit.org’s smokers by recruitment method (Google advertisement, medical practice referral, or dental practice referral, see Table 2). Because Google is frequently the recruitment source for Web-assisted tobacco interventions, we consistently use it as the reference or comparison group.

**Table 1:** Characteristics of smokers participating in the Behavioral Risk Factor Surveillance Survey (BRFSS) and all smokers (Google, medical-referred, and dental-referred combined) who engaged in the Web-assisted tobacco intervention Decide2Quit.org.

Demographic characteristic		Decide2Quit.org combined, n (%) n=604	BRFSS, (%) ^a^ n=69,992
**Sex** ^b^			
	Male	210 (34.7)	(54.3)
	Female	394 (65.2)	(45.7)
**Age** ^b^			
	19-34	116 (19.2)	(33.0)
	35-54	307 (50.8)	(44.1)
	> 55	181 (30.0)	(22.9)
**Race** ^b^			
	White	529 (87.6)	(80.7)
	Nonwhite	75 (12.4)	(19.3)
**Highest grade of school** ^b^			
	< High school	36 (6.0)	(15.7)
	High school	171(28.3)	(38.5)
	Some college or college graduate	397 (65.7)	(45.9)
**During the past 12 months, have you stopped smoking for 1 day or longer because you were trying to quit smoking?** ^c^			
	No	277 (45.9)	(40.9)
	Yes	324 (54.1)	(58.8)
**Smoking status**			
	Not thinking about quitting	26 (4.3)	
	Thinking of quitting	413 (68.4)	
	Set a quit date	72 (11.9)	
	Already quit	93 (15.4)	

^a^Weighted for complex survey design

^b^ *P*=.001

^c^ *P*=.01

**Table 2:** Demographic characteristics and readiness to quit of smokers by recruitment source.

Demographic characteristic		Recruitment source, n (%)		
		Google advertisement n=197	Dental practice referrals n=127	Medical practice referrals n=280
**Sex**				
	Male	61 (31.0)	43 (33.9)	106 (37.9)
	Female	136 (69.0)	84 (66.1)	174 (62.1)
**Age (years)**				
	19-34	29 (14.7)	40 (31.5)	47 (16.8)^a^
	35-54	105 (53.3)	59 (46.5)	143 (51.1)
	< 55	63 (32.0)	28 (22.1)	90 (32.1)
**Race**				
	White	171 (86.8)	112 (88.2)	246 (87.9)
	Nonwhite	26 (13.2)	15 (11.8)	34 (12.1)
**Highest grade of school**				
	< College graduate	134 (68.0)	91 (71.7)	236 (84.3)^b^
	College graduate	63 (32.0)	36 (28.4)	44 (15.7)
**Smoking status**				
	Not thinking about quitting	6 (3.1)	11 (8.7)^b^	9 (3.2)^b^
	Thinking of quitting	112 (56.9)	92 (72.4)	209 (74.6)
	Set a quit date	29 (14.7)	13 (10.2)	30 (10.7)
	Already quit	50 (25.4)	11 (8.7)	32 (11.4)

^a^ *P*=.001 comparing Google and the applicable column

^b^ *P*<.001 comparing Google and the applicable column

### Demographics by Recruitment Source

Demographic and smoking characteristics varied by recruitment source (Table 2). Compared with Google and medical-referred smokers, the dental-referred smokers (21.0%) were younger (*P*=.001 and *P*=.002, respectively). Compared with Google (32.0%) and dental-referred (28.4%) smokers, a lower proportion of medical-referred smokers (15.7%) were college graduates (*P*<.001 and *P*=.003, respectively).

### Readiness to Quit and Other Smoking Characteristics by Recruitment Source

A lower proportion of medical-referred (22.1%) and dental-referred (18.9%) smokers had set a quit date or had already quit than Google-referred smokers (40.1 %, *P*<.001 for both comparisons). The mean number of cigarettes smoked per day was similar between Google (mean 17.8, SD 10.5) and medical-referred smokers (mean 17.4, SD 9.2), but lower for dental-referred smokers (mean 14.5, SD 8.9; *P*=.002). Fewer dental-referred smokers allowed smoking at home compared with the other 2 groups (dental-referred 34% vs Google 48%, *P*=.36; dental-referred 34% vs medical-referred 45%, *P*=.005). Medical-referred (8.6%) and dental-referred (15.8%) smokers were less likely to have visited smoking cessation websites as compared to Google smokers (40.1%, *P*<.001 for both comparisons).

### Smokers’ Participation in Decide2Quit.org by Recruitment Source

Medical-referred (mean 2.4, SD 3.4) and dental-referred (mean 2.1, SD 2.6) smokers visited Decide2Quit.org less frequently than Google smokers (mean 2.7, SD 4.0), but this was not statistically significant (*P*=.14 and *P*=.06, respectively). On average, they also visited fewer pages on the website per visit (medical-referred: mean 12.9, SD 13.6; dental-referred: mean 12.3, SD 12.4; Google: mean 17.4, SD 15.2; Google vs medical-referred *P*<.001; Google vs dental-referred *P*=.002). Compared with Google smokers (42.6%), a lower proportion of medical-referred (29.6%, *P*=.01) and dental-referred (22.8%, *P*=.01) smokers messaged the TTS at least once. Although not statistically significant, among those who messaged at least once, medical- and dental-referred smokers also interacted with the TTS less frequently than their Google counterparts (medical-referred: mean 2.0, SD 2.4; dental-referred: mean 2.0, SD 1.7; Google: mean 3.3, SD 6.1). However, this was not statistically significant (Google vs medical *P*=.05, Google vs dental *P*=.13). Medical-referred (23.6%) and dental-referred (12.6%) smokers also used the online support group less frequently than Google smokers (39.1%, *P*=.01 for both comparisons).

### Multivariable Comparisons Among Google, Medical-Referred, and Dental-Referred Smokers

After adjustment for demographic characteristics and readiness to quit, Google smokers had higher predicted counts of Web page visits (IRR 17.0, 95% CI 15.0-19.1) compared with the medical-referred (IRR 12.7, 95% CI 11.4-14.0) and dental-referred smokers (IRR 12.0, 95% CI 10.1-13.8) (Table 3). Google smokers were also more likely to use the TTS and an online support group. When we assessed system use using number of visits to the website, the direction and magnitude of the point estimates remained the same.

**Table 3:** Associations between recruitment source and use of Decide2Quit.org.

Demographic characteristic		Unadjusted		Adjusted	
		IRR (95% CI)	Counts^a^ (95% CI)	IRR (95% CI)	Counts^a^ (95% CI)
**Patient origin**					
	From Google advertisement	Reference	17.4 (15.3-19.6)	Reference	17.0 (15.0-19.1)
	From medical provider	0.7 (0.6-0.9)	12.9 (11.5-14.2)	0.8 (0.7-0.9)	12.7 (11.4-14.0)
	From dental provider	0.7 (0.6-0.9)	12.3 (10.4-14.2)	0.7 (0.6-0.9)	12.0 (10.1-13.8)
**Sex**					
	Female	Reference	15.3 (13.9-16.6)	Reference	14.8 (13.6-16.1)
	Male	0.8 (0.7-0.9)	12.3 (10.8-13.8)	0.8 (0.7-1.0)	12.1 (10.7-13.5)
**Age**					
	19-34	Reference	14.5 (12.7-16.9)	Reference	14.3 (12.0-16.6)
	35-54	1.0 (0.8-1.2)	13.8 (12.4-15.2)	0.9 (0.8-1.1)	13.4 (12.1-14.7)
	> 55	1.0 (0.82-1.25)	14.7 (12.8-16.7)	1.0 (0.8-1.2)	14.3 (12.5-16.1)
**Race**					
	White	Reference	11.7 (9.3-14.1)	Reference	11.5 (9.2-13.9)
	Nonwhite	1.3 (1.0-1.6)	14.6(13.5-15.7)	1.2 (1.0-0.5)	14.2 (13.1-15.2)
**School**					
	College graduate	Reference	18.4 (15.8-21.1)	Reference	17.8 (15.3-20.4)
	< College graduate	0.7 (0.6-0.8)	13.0 (11.9-14.0)	0.7 (0.6-0.9)	12.8 (11.8-13.8)
**Smoking status**					
	Already quit	Reference	16.2(13.3-19.1)	Reference	15.9 (13.1-18.7)
	Set a quit date	0.9 (0.7-1.2)	15.1 (12.0-18.3)	1.0 (0.8-1.3)	14.9 (11.9-17.9)
	Thinking of quitting	0.9 (0.7-1.1)	13.9 (12.7-15.1)	0.9 (0.8-1.1)	13.5 (12.4-14.6)
	Not thinking about quitting	0.6 (0.4-0.9)	9.9 (6.4-13.4)	0.7 (0.5-1.0)	10.1 (6.6-13.5)

^a^Counts are marginal predicted counts products postregression using the X command in STATA.

## Discussion

### Findings and Conclusions

Approximately 70% of the 44.5 million adult smokers in the United States want to quit, but fewer than 5% of those who do try to quit in a given year succeed [6]. Thus, expanding the reach of effective treatments, such as Web-assisted tobacco interventions, is crucial in increasing quit rates. However, recruitment to Web-assisted tobacco interventions poses unique challenges [22,24-26]. In this study, we expanded recruitment by adding an e-referral approach to the traditional search engine method. In this paper, we assessed how this combination increased the variability of our cohort.

Compared with the population of smokers responding to the 2010 BRFSS, a higher proportion of smokers registering with Decide2Quit.org were female (nearly 20% more). They also were more likely to identify themselves as white in race/ethnicity and be highly educated. However, among those recruited from medical practices, 15.7% reported college education, which was the same proportion reported by BRFSS-participating smokers. Inconsistent with expectations, Decide2Quit.org smokers were older. The rate of prior quit attempts in the past 12 months was similar among those registering with Decide2Quit.org and the national BRFSS.

Our comparisons (as shown in Table 1) highlight the sharp difference in the proportion of women participating in our Web-assisted tobacco intervention as compared with the national sample of smokers. The higher proportion of women also was consistent across our recruitment sources (Google advertisement 69.0% vs medical referral 62.1% vs dental referral 66.1%). Women, in general, may be more likely to participate in a Web-assisted tobacco intervention; therefore, our participation rates may just be reflecting general trends. Across 6 other Web-assisted tobacco interventions, the mean proportion of women participating was 60% (range 52-72) [39-44]. The lower participation rates of men in these interventions suggest that different recruitment approaches or different types of interventions might be needed to engage them in cessation activities.

Although we identified differences in race/ethnicity and education for our Web-engaged smokers compared to the BRFSS, our age distribution was older than the national sample. The older age may be because the decision to make a serious and successful attempt to quit smoking is typically made when a smoker has reached a greater level of maturity than the average smoker (mid- to upper-30s). Other Web-assisted tobacco interventions also report a mean age of 39 years (range 34-49, [39-44]). However, close to one-third of our smokers were over the age of 55. This number may be reflective of the decrease in age barriers to Internet adoption in recent years. [45].

Recruitment from medical and dental practices was more efficient than Google advertising with respect to the number of referrals needed to register a smoker (5.7, 5.8, and 35.5, respectively). Within the population of smokers registering at Decide2Quit.org, the types of smokers who were recruited from clinical practices were different from those who found the intervention site via Google. The general trend in educational status among our Web-engaged smokers varied. Those recruited from medical practices were less educated and less likely to have previously used a Web-assisted tobacco intervention; thus, they more closely resembled the national sample. Dental practice smokers were younger than counterparts from the other recruitment groups. Many younger smokers are seen in dental practices, but may not be seen in medical practices; thus, recruitment from dental practices allowed us to target smokers who were not engaged through Google or medical practices. These dental participants also were lighter smokers, a group that may be especially difficult to engage in interventions to quit smoking.

In addition to engaging smokers with different demographic characteristics, clinical practice-based recruitments also resulted in participation by smokers with a wider range of motivational levels. Our results support previous concerns that recruiting using search engine-based recruitment methods alone may limit the reach of Web-assisted tobacco interventions mainly to smokers highly motivated to quit [46-50]. Currently, most smokers recruited to these interventions are through search engine advertisements or other mass media campaigns, which require the smoker to be motivated to register on the system. For example, out of 2523 smokers recruited to a Web-assisted tobacco intervention [27], most (71%) were recruited through Google Ads or direct mailing. Only 95 smokers (3.8%) were recruited through provider referrals or other proactive recruitment methods. The recruitment to the National Colorectal Cancer Research Alliance (NCCRA) and OncoLink Web-assisted tobacco intervention [28] also were primarily through search engine and mass media campaigns. In the OncoLink study, only 7.3% of 2162 smokers were registered from proactive provider referrals.

As noted, our results indicate that using only search engine or mass media recruitment methods limits the range of smokers engaged. In our study, a higher proportion of Google smokers were ready to quit or had already quit compared with other smokers; medical practice referrals brought in smokers who were often at a lower readiness to quit and less likely to have sought help from online resources. They are an important group of smokers to engage, and Web-assisted tobacco interventions can be designed for smokers not ready to quit (as cessation induction interventions) as well as for smokers ready to quit (as an aid to cessation).

Although we were successful in broadening our sample, we were unable to maintain the engagement of clinical practice-recruited smokers at the same level as Google-recruited smokers. Google participants had higher participation rates at Decide2Quit.org. Previous Web-assisted tobacco intervention studies have shown a relationship between smoking cessation and number of website visits [51], number of website sections viewed [52], and amount of time spent on the website [53]. Thus, Google participants may have disproportionally benefited from the intervention. Google smokers had a higher number of page hits, even after adjusting for demographics and readiness to quit. Google smokers were more educated, had prior experience participating in a Web-assisted tobacco intervention, and were more likely to have set a quit date or quit. Other important predictors of greater use included TTS use and access to an external social network. Future Web-assisted tobacco interventions may need to be flexible in their strategy to maintain engagement for smokers not quite ready to quit [54], perhaps by continually monitoring participation rates and programming their interventions to be more proactive with the groups that are less engaged.

### Limitations

Our study has limitations. First, we collected a limited number of characteristics of these smokers; thus, the samples may vary on important unmeasured characteristics. Some of the information was self-reported through an online survey and cannot be validated. We evaluated the impact of only 1 Web-assisted tobacco intervention (Decide2Quit.org), which prevents strict generalizability to all Web-assisted tobacco interventions or other online behavior support. One major difference in participation in the intervention was by readiness to quit. Although, we adjusted for readiness to quit in our model (Table 3), it is certainly possible that residual confounding by readiness is mediating differences in participation by recruitment source.

In conclusion, to maximize the potential of Web-assisted tobacco interventions, expanding methods to attract more smokers is critical. In recruiting users who typically do not participate in these interventions, we demonstrated that clinical practice recruitment does complement Internet search engine recruitment. However, our results also suggest that once recruited, those smokers recruited from clinical practices may not be as active as the Google smokers, suggesting that Web-assisted tobacco interventions may need to tailor their engagement strategies.

## Abbreviations

BRFSS: Behavioral Risk Factors Surveillance Survey
IRR: incidence rate ratio
TTS: tobacco treatment specialist
